# A Comprehensive Review of Postoperative Analgesics Used in Orthopedic Practice

**DOI:** 10.7759/cureus.48750

**Published:** 2023-11-13

**Authors:** Sachin Goel, Sanjay V Deshpande, Vivek H Jadawala, Anmol Suneja, Rahul Singh

**Affiliations:** 1 Orthopaedics, Jawaharlal Nehru Medical College, Datta Meghe Institute of Higher Education and Research, Wardha, IND

**Keywords:** postoperative pain management, adverse effects management, patient-centered care, emerging trends, multimodal analgesia, orthopedic surgery

## Abstract

Orthopedic surgeries, ranging from joint replacements to fracture fixations, are integral procedures that enhance the quality of life for countless individuals. Effective postoperative pain management is crucial in ensuring optimal patient recovery and satisfaction. This comprehensive review analyzes the diverse array of analgesic modalities employed in orthopedic practice for postoperative pain relief. The review systematically explores the pharmacological landscape of analgesics commonly used in orthopedic settings, including opioids, non-steroidal anti-inflammatory drugs, acetaminophen, and adjuvant medications. Emphasis is placed on their mechanisms of action, efficacy profiles, and potential adverse effects. Special attention is given to the evolving role of multimodal analgesia, which combines various agents to achieve synergistic pain control while minimizing individual drug-related complications. Furthermore, the review addresses the emerging trends and advancements in postoperative analgesia within orthopedics, such as integrating regional anesthesia techniques, peripheral nerve blocks, and novel pharmacological agents. A critical evaluation of evidence-based practices and recent clinical trials is incorporated to guide practitioners in making informed decisions regarding postoperative pain management. Consideration is also given to the individualized nature of pain experiences and the importance of patient-centric approaches. The review underscores the significance of tailoring analgesic regimens based on patient characteristics, surgical procedures, and potential complications, fostering a personalized and effective pain management strategy. In conclusion, this comprehensive review is valuable for orthopedic practitioners, anesthetists, and healthcare professionals involved in postoperative care. By synthesizing current knowledge and highlighting evolving trends, the review contributes to the ongoing dialogue on optimizing pain management strategies in orthopedic practice, ultimately improving patient outcomes and satisfaction.

## Introduction and background

Orthopedic surgery is a specialized branch of medicine that focuses on diagnosing and treating musculoskeletal disorders and injuries, encompassing a wide range of procedures, ranging from joint replacements to fracture repairs. In the context of orthopedic surgery, postoperative pain management is of paramount importance. Patients undergoing orthopedic procedures often experience significant pain due to the nature of the surgeries, which involve bones, joints, and soft tissues. This pain can hinder recovery, impair quality of life, and potentially lead to chronic pain if not effectively managed [1]. Postoperative pain following orthopedic surgeries is multifaceted, influenced by various factors such as the surgical approach, the extent of tissue manipulation, and individual patient characteristics. Patients may experience acute pain, inflammation, and muscle spasms, making effective analgesia a critical aspect of their care. Failing to manage postoperative pain adequately can lead to delayed mobilization, decreased functional outcomes, and prolonged hospital stays [2].

Effective postoperative analgesia in orthopedic practice has profound implications for patient outcomes and satisfaction. Proper pain management enhances patient comfort and facilitates early mobilization, physical therapy, and a faster return to daily activities. It can significantly reduce the risk of postoperative complications and improve overall recovery, making it an essential element of patient care in orthopedic surgery [3]. Moreover, uncontrolled pain can contribute to increased healthcare costs, as patients may require more interventions, more extended hospital stays, and increased pharmaceutical regimens to manage the consequences of inadequately controlled pain. This makes the financial implications of suboptimal analgesia a concern for healthcare systems and institutions [4].

This comprehensive review aims to delve into the multifaceted domain of postoperative analgesics used in orthopedic practice. This review aims to explore the various pharmacological and non-pharmacological methods used to manage postoperative pain in orthopedic patients. It will also examine the evolving trends and emerging innovations in the field. The scope of this review encompasses an extensive analysis of the types of orthopedic surgeries that often require postoperative analgesia, the goals of pain management in orthopedic practice, and the pharmacological analgesic agents commonly employed. In addition to traditional opioid and non-opioid medications, the review will also explore the role of regional anesthesia techniques, such as epidural analgesia and peripheral nerve blocks, and the concept of multimodal analgesia.

## Review

Goals of postoperative analgesia in orthopedic practice

Pain Management Objectives

Pain relief: Pain relief is the fundamental objective of postoperative analgesia. Its primary aim is to alleviate the patient’s suffering by diminishing pain intensity to a manageable and bearable level. Effective pain relief ensures that patients can move, breathe, and engage in rehabilitation activities comfortably. By reducing pain to tolerable levels, patients can focus on their recovery and well-being rather than being burdened by severe discomfort [5].

Facilitate early mobilization: Early mobilization is critical to postoperative care, and effective pain management is pivotal in enabling it. Patients who are experiencing severe pain may be reluctant to move or engage in physical activities, which can lead to a higher risk of complications such as deep vein thrombosis, atelectasis, and pressure ulcers. Adequate pain control allows patients to mobilize sooner, promoting better circulation and lung function and preventing these potentially severe postoperative complications [6].

Enhance surgical outcomes: The impact of pain control extends beyond mere comfort; it can significantly influence the overall success of the surgical procedure. Patients who are not plagued by severe pain are more likely to adhere to their postoperative care plans, which often include crucial components such as physical therapy and rehabilitation exercises. By actively participating in these rehabilitation activities, patients can enhance their surgical outcomes, regain functional mobility more quickly, and achieve better long-term results [5].

Minimize adverse effects: Effective pain management is not solely about pain relief; it also entails minimizing the potential adverse effects of the analgesic agents. This includes managing and mitigating the risks associated with opioid analgesics, such as respiratory depression and constipation. Striking a balance between pain control and avoiding side effects is essential to ensure patient safety and comfort [7].

Improve patient satisfaction: Patient satisfaction is vital to postoperative care. A positive patient experience can significantly influence their perception of their care and overall satisfaction with the healthcare facility. Effective pain management is a central factor in determining patient satisfaction. When patients experience successful pain relief and comfort during their recovery, they are more likely to view their healthcare experience favorably, leading to improved overall outcomes and a positive reputation for the healthcare institution [8].

Factors Influencing the Choice of Analgesic Agents

Type of surgery: The type of orthopedic surgery is a pivotal determinant in selecting appropriate analgesic agents. Major joint replacement surgeries, such as hip or knee replacements, typically necessitate a more robust and comprehensive pain management approach, often involving opioids and regional anesthesia techniques. In contrast, minor arthroscopic procedures may permit the use of less potent analgesics, such as non-steroidal anti-inflammatory drugs (NSAIDs), to achieve adequate pain control while minimizing the potential for opioid-related side effects [9].

Patient characteristics: Patient-specific factors are instrumental in tailoring analgesic choices. Age is a critical consideration, as elderly patients may be more susceptible to opioid-related side effects, necessitating dose adjustments or alternative agents. Furthermore, patients with comorbid conditions, such as cardiovascular diseases or respiratory disorders, may require careful selection of analgesics to avoid exacerbating their underlying health issues. Allergies and sensitivities must be meticulously addressed to prevent adverse reactions, and alternative agents must be identified for patients with a history of drug allergies [10].

Pain intensity: The severity of postoperative pain experienced by the patient is a primary factor in determining the choice of analgesic agents. The intensity of pain often dictates the need for more potent analgesics. Mild pain, which is common after less invasive orthopedic procedures, may be effectively managed with non-opioid analgesics such as NSAIDs and acetaminophen. In contrast, severe pain, frequently encountered after significant surgeries, often necessitates the use of opioids to provide adequate pain relief [11].

Risk of adverse effects: The potential for adverse effects associated with specific analgesic agents is a critical consideration in pain management. Healthcare providers must balance pain control and avoiding or mitigating side effects. Opioids, for instance, are highly effective in alleviating severe pain but carry a risk of respiratory depression and constipation. Careful monitoring and dose adjustments are essential to minimize these risks [7].

Patient preferences: Patient input and preferences are integral to decision-making. Some patients may have strong preferences regarding the analgesic they receive. For instance, some may wish to avoid opioids due to concerns about sedation and constipation. In contrast, others may have had positive experiences with opioids in the past and feel more comfortable with this type of medication [12,13].

Balanced Approach to Analgesia

Individualized care: Individualized care in pain management is paramount in tailoring treatments to each patient’s unique needs and characteristics. Recognizing that pain experiences vary among individuals, healthcare providers employ a personalized approach to select the most appropriate analgesic agents, doses, and administration routes for each patient. This strategy ensures patients receive the most effective pain relief while minimizing adverse effects, ultimately improving patient outcomes and satisfaction [14].

Multimodal analgesia: The concept of multimodal analgesia involves the judicious combination of various analgesic agents and techniques to address pain through multiple pathways and mechanisms. By diversifying the approaches to pain management, healthcare providers reduce the reliance on opioids, which are associated with numerous side effects and potential risks. Multimodal analgesia aims to maximize pain control while minimizing the adverse effects of any single agent, resulting in a more balanced and effective postoperative pain management strategy [15].

Titration and monitoring: The titration and continuous monitoring of analgesic regimens are integral to effective pain management. This approach involves assessing and adjusting the analgesic regimen in response to evolving pain levels and patient responses. It ensures that patients receive the optimal dose of analgesics, neither undermedicating nor overmedicating, thereby achieving the best balance between pain control and safety [16].

Patient education: Patient education is crucial to pain management. Patients must be informed about their pain management options, the potential side effects of analgesic agents, and the importance of open communication with the healthcare team. Educating patients empowers them to actively participate in their pain management, allowing them to make informed decisions and express their preferences. An informed patient is more likely to have a positive experience and better adherence to the prescribed pain management plan [17].

Early intervention: Addressing pain as soon as it arises is a proactive approach that can prevent it from escalating to severe levels. Early intervention involves recognizing the signs of pain, promptly administering analgesic agents or techniques, and monitoring the patient’s response. By taking swift action, healthcare providers can effectively manage pain and minimize its impact on the patient’s comfort and recovery, ultimately leading to better postoperative outcomes [18].

Pharmacological analgesic agents

Non-opioid Analgesics

Nonsteroidal anti-inflammatory drugs*: *NSAIDs exert their therapeutic effects by inhibiting the activity of cyclooxygenase (COX) enzymes. These enzymes catalyze arachidonic acid’s conversion into prostaglandins, critical inflammation, pain, and fever mediators. NSAIDs interfere with this process by blocking COX enzymes, thereby reducing the production of prostaglandins. As a result, inflammation and pain are diminished, making NSAIDs valuable in managing orthopedic postoperative pain [19]. NSAIDs are frequently used in orthopedic practice, especially in managing mild-to-moderate postoperative pain and inflammation following orthopedic procedures. They are particularly effective for conditions where inflammation plays a significant role in pain generation, such as osteoarthritis, tendonitis, or postsurgical inflammatory responses. NSAIDs are often integrated into multimodal analgesia regimens, aiming to reduce the reliance on opioid analgesics. By combining NSAIDs with other analgesic agents, healthcare providers can provide more balanced pain relief while minimizing the risks and side effects associated with higher doses of opioids. This approach enhances pain control and contributes to patient comfort and satisfaction during the recovery process in orthopedic practice [19].

Acetaminophen (paracetamol): Acetaminophen, also known as paracetamol, primarily exerts its mechanism of action centrally in the brain. It acts as an antipyretic and analgesic agent by inhibiting the synthesis of prostaglandins within the central nervous system, specifically in the hypothalamus, which plays a crucial role in regulating body temperature and pain perception. By reducing the production of prostaglandins in the brain, acetaminophen effectively lowers body temperature (antipyresis) and relieves pain [20]. Acetaminophen is a widely used non-opioid analgesic, often serving as a first-line option for mild-to-moderate pain management. It is precious in cases where patients may not tolerate NSAIDs due to gastrointestinal issues, such as ulcers or bleeding risks. Acetaminophen is known for its relatively low incidence of gastrointestinal side effects, making it a favorable choice for patients with preexisting digestive conditions or those at risk of bleeding complications. It is typically administered orally and can be a crucial component of multimodal analgesia regimens to minimize the reliance on opioids, contributing to adequate pain control while reducing the potential for opioid-related side effects in orthopedic postoperative care [20].

Opioid Analgesics

Morphine: Morphine is a potent opioid analgesic that exerts its primary mechanism of action by interacting with μ-opioid receptors in the central nervous system. By binding to these receptors, morphine modulates the perception of pain, leading to a reduction in the intensity of pain experienced by the patient. This mechanism also contributes to the sedative and analgesic effects of morphine [21]. In orthopedic practice, morphine is typically reserved for the management of severe postoperative pain that is unresponsive to non-opioid analgesics. While non-opioid analgesics such as NSAIDs and acetaminophen are often effective for mild-to-moderate pain, they may not provide adequate relief for more intense pain following major orthopedic surgeries, such as joint replacements or spinal procedures. Morphine, as an opioid, is particularly valuable in these cases [21].

Oxycodone: Oxycodone is a semi-synthetic opioid analgesic that primarily functions as a μ-opioid receptor agonist. By binding to μ-opioid receptors in the central nervous system, oxycodone exerts its analgesic effects, modulating pain perception. This mechanism enables oxycodone to provide adequate pain relief, making it a valuable option for managing moderate-to-severe pain [22]. Oxycodone is frequently employed in orthopedic practice to manage moderate-to-severe pain following surgical procedures. It is particularly beneficial in cases where non-opioid analgesics such as NSAIDs and acetaminophen may not provide adequate pain control. Oxycodone is often administered in combination with other analgesic agents as part of a multimodal approach to postoperative pain management. This approach aims to enhance pain relief while minimizing the reliance on high doses of any single medication, thereby reducing the risk of opioid-related side effects and complications [22].

Hydrocodone: Hydrocodone is a semi-synthetic opioid analgesic that shares its mechanism of action with other opioids. It primarily provides analgesia by binding to μ-opioid receptors in the central nervous system. By interacting with these receptors, hydrocodone modulates the perception of pain, resulting in effective pain relief [23]. Hydrocodone is employed in orthopedic practice to manage acute postoperative pain, particularly in cases where non-opioid analgesics alone may not suffice to provide adequate pain control. It is often used in combination with non-opioid analgesics, such as NSAIDs or acetaminophen, as part of a multimodal approach to postoperative pain management [23].

Adjuvant Analgesics

Gabapentin and pregabalin: Gabapentin and pregabalin are anticonvulsant medications that exert their analgesic effects through a different mechanism than traditional analgesics. They modulate calcium channels in the central nervous system, which reduces the release of excitatory neurotransmitters, such as glutamate. By diminishing the release of these neurotransmitters, gabapentin and pregabalin help alleviate neuropathic pain, which is characterized by abnormal nerve signaling and sensitivity [24]. Gabapentin and pregabalin are increasingly utilized as adjuvant analgesics in orthopedic practice, especially for the management of neuropathic pain that may occur postoperatively. Neuropathic pain can be a challenging aspect of orthopedic surgery, often resulting from nerve compression, injury, or irritation during the procedure. These anticonvulsant agents can be valuable additions to postoperative pain management regimens as they target the specific mechanisms underlying neuropathic pain [24].

Tricyclic Antidepressants

Tricyclic antidepressants (TCAs) are a class of medications that, in the context of pain management, exert their effects by inhibiting the reuptake of certain neurotransmitters, namely, serotonin and norepinephrine. TCAs can modulate pain perception and may contribute to pain relief by prolonging the presence of these neurotransmitters in the synaptic cleft [25]. TCAs are sometimes employed for their analgesic properties in orthopedic practice, particularly for the management of chronic pain conditions, including neuropathic pain that can result from orthopedic procedures. Neuropathic pain is characterized by abnormal signaling of the nervous system and is often resistant to traditional analgesics. TCAs may be used to address this type of pain [25].

Anticonvulsants

Certain anticonvulsant medications, such as carbamazepine and lamotrigine, exert their analgesic effects by modulating neuronal excitability in the central nervous system. These drugs influence the transmission of signals between nerve cells and can help manage conditions characterized by abnormal nerve signaling, such as neuropathic pain [26]. Anticonvulsants are occasionally employed in postoperative care, although not as commonly used as other analgesics. They may be considered when neuropathic pain is a significant component of a patient’s postoperative discomfort, mainly when traditional analgesics such as opioids, NSAIDs, or acetaminophen are insufficient in providing relief [26].

Regional anesthesia techniques

Epidural Analgesia

Epidural analgesia is known for its effectiveness in providing pain relief. It involves the administration of local anesthetic medications into the epidural space surrounding the spinal cord. This results in a highly targeted and comprehensive numbing effect on the nerves in the lower half of the body, making it particularly suitable for procedures such as childbirth or postoperative pain management. Adequate pain control enables patients to remain comfortable, which is essential for their well-being and allows for early mobilization. Patients not in significant pain are more likely to participate in activities promoting their recovery [27].

One of the significant advantages of epidural analgesia is its ability to reduce the need for systemic opioids. Opioids, when administered systemically (e.g., intravenously or orally), can lead to side effects such as drowsiness, nausea, constipation, and a risk of opioid dependency. Epidural analgesia, by targeting pain at the spinal level, minimizes the systemic distribution of opioids, thereby reducing the risk of these side effects. This can be especially important in the context of postoperative recovery, where patients need effective pain control without the drawbacks of excessive opioid use [27].

Epidural analgesia can positively impact pulmonary function by reducing pain-related respiratory complications. Severe pain after surgery can lead to shallow breathing, reduced lung expansion, and an increased risk of complications such as atelectasis (partial lung collapse) and pneumonia. With epidural analgesia, patients experience better pain control, allowing them to take deeper breaths and cough effectively. This can help prevent respiratory issues and promote overall lung function, which is particularly beneficial for patients undergoing chest or abdominal surgeries [28].

Peripheral Nerve Blocks

Peripheral nerve blocks are designed to provide precise and localized pain relief to specific body areas. They achieve this by injecting anesthetic medications directly around the nerves responsible for transmitting pain signals in the targeted region. This focused approach allows healthcare providers to effectively numb or block pain in a particular body part, such as an arm, leg, or abdominal region, without affecting the entire body. This targeted pain relief is especially beneficial in cases where systemic analgesics might lead to unwanted side effects or where localized pain management is essential [29].

Peripheral nerve blocks can significantly reduce the need for systemic opioids, such as morphine or oxycodone. By directly blocking pain signals at the nerve level, these blocks can provide adequate pain control, often without the need for high doses of opioids. This is particularly important in the context of the opioid epidemic, as it helps to minimize the risks of opioid addiction, respiratory depression, and other side effects associated with opioid use. It also allows patients to experience less sedation and greater clarity of mind, which can be essential for postoperative recovery [30].

Effective pain control through peripheral nerve blocks can promote early mobilization, physical therapy, and rehabilitation. When patients experience less pain, they are more likely to engage in activities that aid their recovery. Early rehabilitation is associated with faster recovery times, improved functional outcomes, and a reduced risk of complications such as muscle atrophy or blood clots. More comfortable patients can also perform deep breathing exercises, cough effectively, and ambulate, which can be crucial in preventing postoperative complications [31].

Intrathecal Analgesia

Intrathecal administration involves injecting medication directly into the space surrounding the spinal cord, allowing for rapid onset of pain relief. This is particularly advantageous for postoperative pain management, as it can quickly alleviate discomfort and improve the patient’s comfort. When medication is introduced into the cerebrospinal fluid through intrathecal injection, it bypasses the digestive system and bloodstream, reaching the spinal cord and brain more directly. As a result, pain relief is faster than oral or parenteral (e.g., intravenous) administration, which may have a delayed onset due to the time required for absorption and distribution in the body [32].

One of the significant advantages of intrathecal analgesia is its ability to minimize systemic side effects associated with other methods of medication administration. When administered orally or intravenously, medications circulate throughout the body, affecting various organs and systems. This can lead to unwanted side effects such as nausea, vomiting, constipation, or respiratory depression, which are often seen with opioid analgesics. On the other hand, Intrathecal administration delivers the medication directly to the cerebrospinal fluid, where it acts locally on the nerves involved in pain transmission. This localized action reduces the likelihood of systemic side effects, making it a safer and more comfortable option for patients [33].

Intrathecal analgesia provides the advantage of precise and controlled pain relief duration. Healthcare professionals can titrate the medication to meet the patient’s needs during the critical postoperative period. This level of control ensures that the patient receives consistent and predictable pain management without the risk of under or overmedication. Adjusting the dosage and duration of pain relief allows for a tailored approach to patient care, minimizing the risk of excessive sedation or inadequate pain control [34].

Multimodal analgesia

Rationale for Multimodal Analgesia

Pain is a complex phenomenon with various pathways and mechanisms involved. Multimodal analgesia recognizes this complexity and employs different analgesic agents that target distinct components of the pain pathway. Doing so provides a more comprehensive and practical approach to pain relief. Each agent in the multimodal regimen can address specific aspects of the pain experience, resulting in more thorough pain control. This approach is precious in orthopedic practice, where patients may experience a range of pain types, including nociceptive and neuropathic pain [35].

Over-reliance on opioids for pain management can lead to a range of adverse consequences, including opioid-related side effects, addiction, and the potential for involvement in the opioid crisis. Multimodal analgesia aims to reduce the need for high-dose opioids by incorporating non-opioid analgesics and techniques. This reduction in opioid reliance promotes safer pain management while still achieving effective pain control. It aligns with the broader healthcare goal of minimizing the opioid epidemic’s impact and provides a more balanced and cautious approach to pain relief in orthopedic practice [36].

Multimodal analgesia leverages the strengths of multiple analgesic agents with different mechanisms of action. Combining these agents can provide pain relief more effectively than using a single agent alone. This approach is especially advantageous for patients with severe or complex pain, such as those undergoing major orthopedic surgeries. Multimodal analgesia ensures that different aspects of the pain experience are addressed simultaneously, optimizing pain control and enhancing patient comfort during the postoperative period [15].

Multimodal analgesia is patient-centered and allows for personalized pain management. Each patient’s pain experience is unique, and a multimodal approach can be adjusted to address their specific needs, preferences, and sensitivities. This personalized care ensures that patients receive the most suitable combination of analgesic agents and techniques, aligning with the principles of individualized pain management. Tailored pain management enhances patient satisfaction, promotes engagement in their care, and contributes to better postoperative outcomes in orthopedic practice [37].

Combination Therapy Using Various Analgesic Agents

Non-opioid analgesics, such as NSAIDs and acetaminophen, serve as the foundational components of a multimodal approach to pain management. These agents are effective in addressing inflammation and managing mild-to-moderate pain. They are often used to reduce the reliance on opioids, promoting safer and more balanced pain control. In orthopedic practice, NSAIDs and acetaminophen can enhance patient comfort, particularly during the early stages of recovery [15].

Opioid analgesics, including medications such as morphine, oxycodone, and hydrocodone, have a place in the multimodal approach to pain management for more severe pain. However, when integrated into multimodal regimens, they are typically administered at lower doses than if used as the sole analgesic. This approach aims to provide adequate pain control while minimizing the risk of opioid-related side effects, such as respiratory depression and constipation. Opioid use in multimodal analgesia is carefully considered, and dosages are adjusted to achieve the desired pain relief with a reduced likelihood of adverse effects [35].

Regional anesthesia techniques, such as epidural analgesia, peripheral nerve blocks, and intrathecal analgesia, can be seamlessly integrated into the pain management plan to target specific pain areas and enhance overall pain control. These techniques provide a localized approach to pain relief, reducing the need for systemic medications and minimizing side effects. In orthopedic practice, regional anesthesia is particularly valuable for postoperative pain management after procedures involving specific joints or extremities, enabling patients to experience more targeted pain relief [38].

Adjuvant analgesics, which encompass medications such as gabapentin, pregabalin, TCAs, and anticonvulsants, play a vital role in complementing the primary analgesics in multimodal approaches, especially when neuropathic pain is a component of the patient’s discomfort. These medications target specific mechanisms of pain transmission, making them valuable additions to pain management regimens. In orthopedic practice, adjuvant analgesics can be tailored to address neuropathic pain or other pain components that may not respond optimally to traditional analgesics [39].

Benefits and Challenges of Multimodal Analgesia

Benefits of multimodal analgesia: Multimodal analgesia offers the advantage of more comprehensive and effective pain relief than relying on a single analgesic agent. By combining drugs and techniques with different mechanisms of action, healthcare providers can address pain from multiple angles, resulting in enhanced patient comfort and satisfaction. This improved pain control is precious in orthopedic practice, where pain management is pivotal to postoperative recovery and rehabilitation [35].

Multimodal analgesia strategies aim to minimize the reliance on high-dose opioids, thereby reducing the risk of opioid-related side effects. Opioid-related complications, such as respiratory depression, constipation, and the potential for addiction, can be mitigated by incorporating non-opioid analgesics and regional anesthesia techniques. This approach promotes patient safety and well-being by providing adequate pain control while minimizing the adverse effects associated with opioids [36].

Multimodal analgesia improves pain control and facilitates early mobilization and rehabilitation. Patients who experience more comfortable pain relief are more likely to engage in postoperative care plans, such as physical therapy and exercises, which are essential for a speedy recovery. Faster recovery and improved functional outcomes contribute to better overall patient experiences and outcomes in orthopedic practice [15].

A balanced approach to pain management, as achieved through multimodal analgesia, enhances the safety of orthopedic procedures. The reduction in opioid use and the incorporation of alternative analgesics and techniques minimize the risks associated with high-dose opioids. This approach is essential for vulnerable patient populations, such as the elderly and those with preexisting medical conditions, as it reduces the potential for adverse events and complications during the postoperative period [40].

Challenges of Multimodal Analgesia: Implementing complex pain management regimens that involve multiple medications and techniques can be challenging. Healthcare providers must coordinate the administration of different analgesics and monitor their effects closely. Complex regimens require precise dosing schedules and thorough patient education to ensure patients understand how to follow the plan effectively. Comprehensive documentation and communication among healthcare team members are essential to avoid errors and provide optimal care [41].

Patients’ responses to various analgesic agents can differ significantly. Tailoring the pain management regimen to each patient’s unique needs is essential to achieve adequate pain control while minimizing adverse effects. When selecting and adjusting analgesics, healthcare providers must conduct a thorough pain assessment and consider individual factors, such as age, comorbidities, and sensitivities. Personalized care acknowledges that one size does not fit all in pain management and is crucial for optimizing patient outcomes [42].

Using multiple agents and techniques in complex pain management regimens may increase costs. However, these costs are often offset by several factors, such as shorter hospital stays, improved patient outcomes, and reduced complications. Multimodal and individualized approaches to pain management can contribute to quicker recoveries, reduced healthcare resource utilization, and enhanced patient satisfaction. The long-term benefits of improved patient well-being and postoperative outcomes often justify the upfront investment in comprehensive pain management regimens [43].

Patient-specific considerations

Age, Gender, and Pain Perception

Age: Patients’ age plays a significant role in how they perceive and respond to pain and their sensitivity to analgesic agents. Elderly patients often exhibit altered pharmacokinetics, affecting drug absorption, distribution, metabolism, and elimination. They may require lower doses of certain analgesics to prevent adverse effects. Additionally, older patients may experience increased sensitivity to opioids, which can result in a higher risk of side effects, including respiratory depression. Pediatric patients, on the other hand, necessitate age-appropriate dosing and pain assessment techniques to ensure safe and effective pain management. Tailoring analgesia to age-specific needs is crucial to provide optimal care and minimize the risk of adverse effects in both pediatric and elderly populations [44].

Gender: Research has indicated that pain perception and analgesic agent responses vary by gender. Understanding these differences and considering gender-specific factors is essential when developing pain management strategies. For instance, women may experience increased sensitivity to pain, potentially influencing the choice of analgesics and dosages. Healthcare providers should be aware of gender-specific pain responses and adapt pain management plans to account for these variations, ensuring that patients receive personalized and effective care [45].

Coexisting Medical Conditions

Cardiovascular conditions: Patients with preexisting cardiovascular diseases require special consideration when selecting analgesic agents. Some analgesics, such as NSAIDs, can increase the risk of cardiovascular events such as heart attacks and strokes. Therefore, careful evaluation is necessary to choose analgesics that are safe for patients with cardiovascular conditions. When planning pain management, healthcare providers must consider the patient’s specific cardiovascular status, medications, and risk factors [46].

Respiratory conditions: Patients with respiratory disorders, such as chronic obstructive pulmonary disease, may be more vulnerable to the respiratory depressant effects of opioids. Adjusting opioid doses and considering non-opioid analgesics are crucial in these cases to minimize the risk of respiratory depression. Patients’ lung function and oxygenation should be closely monitored to ensure their safety during pain management [47].

Renal and hepatic impairment: Impaired renal or hepatic (liver) function can significantly affect the metabolism and elimination of analgesic agents. Healthcare providers must be diligent in monitoring patients with these conditions, as dosing adjustments are often necessary to prevent drug accumulation and potential toxicity. The choice of analgesics and their specific dosages should be tailored to the patient’s level of impairment to maintain safety and efficacy in pain management [48].

Diabetes: Patients with diabetes may experience altered pain perception and slower wound healing, which can impact their postoperative pain experience. Monitoring blood sugar levels is essential to ensure that diabetes is well-managed during the postoperative period. Additionally, healthcare providers should tailor pain management to account for the patient’s unique needs, taking into consideration both the altered pain perception and the importance of wound healing in diabetic patients. This may involve the use of analgesic agents that do not interfere with blood sugar control and a more cautious approach to pain management to prevent complications [49].

Medication Allergies and Sensitivities

Allergies: Patient allergies are critical in pain management planning. Patients with known drug allergies, including allergies to specific analgesic agents, require careful assessment to avoid administering medications that could trigger allergic reactions. Allergies can range from mild skin rashes to severe anaphylactic reactions, making it imperative to select alternative analgesic agents that do not pose an allergy risk. A thorough review of the patient’s medical history and a comprehensive allergy assessment should guide the selection of safe and suitable analgesics, prioritizing the patient’s safety and well-being [50-52].

Sensitivities: Some patients may exhibit sensitivities or idiosyncratic reactions to certain medications, even if they do not have allergies. These sensitivities can result in unexpected adverse effects or intolerance to specific analgesic agents. Healthcare providers must be aware of such sensitivities, as they may influence the choice of analgesics and the development of individualized pain management regimens. In cases of sensitivities, alternative medications or approaches may be needed to ensure adequate pain control while minimizing the risk of adverse reactions. Careful monitoring and communication with the patient are essential to address sensitivities and tailor pain management strategies accordingly [51,53-55].

Emerging trends and innovations

New Analgesic Agents and Delivery Methods

Novel analgesic agents: Ongoing research and development efforts have yielded a new generation of analgesic agents with innovative mechanisms of action. These agents, which may include highly selective opioid receptor modulators, NMDA receptor antagonists, and ion channel blockers, hold the promise of providing enhanced pain control while minimizing side effects. These advancements are especially valuable in orthopedic practice, where effective pain management is crucial for postoperative recovery. The introduction of novel analgesic agents expands the options available to healthcare providers, allowing them to tailor pain management regimens to each patient’s specific needs and circumstances [56].

Sustained-release formulations: Innovations in drug delivery technology have led to the development of sustained-release formulations for existing analgesic agents. These formulations offer several advantages in orthopedic practice. They provide prolonged pain relief, reducing the need for frequent dosing and simplifying medication regimens for patients. This enhances patient convenience and improves medication adherence, essential for effective pain management. Sustained-release formulations can be particularly beneficial for patients undergoing orthopedic surgeries, as they ensure steady and consistent pain relief throughout the postoperative period [57].

Topical analgesics: Topical analgesics, including creams, gels, and patches, have gained popularity for localized pain control in orthopedic practice. These non-invasive and patient-friendly formulations are beneficial for managing pain associated with specific musculoskeletal conditions such as osteoarthritis. They offer a targeted approach to pain relief, allowing patients to apply the analgesic directly to the affected area, minimizing systemic side effects. The convenience and ease of use make topical analgesics a valuable addition to the pain management options available in orthopedic care [58].

Advances in Pain Assessment and Monitoring

Digital health solutions: Integrating digital health technologies represents a transformative trend in pain management. Smartphone apps and wearable devices have enabled real-time pain assessment and monitoring. Patients can effortlessly report their pain levels and activities, providing healthcare providers with a wealth of data to tailor personalized pain management plans. These digital solutions offer a more patient-centered approach, as they actively engage patients in their pain management and offer healthcare providers a comprehensive view of the patient’s pain experience, ultimately leading to more effective and individualized care [59].

Objective pain measurement: Advances in research are leading to the development of objective measures of pain. Biomarkers and imaging techniques are being explored to provide quantifiable pain intensity and location data. This shift toward objective pain measurement enhances the accuracy of pain assessment and monitoring, reducing the reliance on self-reporting, which can be subjective and influenced by various factors. Objective measures offer healthcare providers a more precise understanding of a patient’s pain experience, allowing for targeted interventions and a better assessment of the effectiveness of pain management strategies [60].

Telemedicine: The growth of telemedicine platforms has revolutionized access to pain management expertise. Patients can now engage in remote pain assessment and consultation with healthcare providers, offering greater convenience, especially for follow-up care. Telemedicine is particularly advantageous for patients in remote or underserved areas, as it provides a means to access specialized care without the need for extensive travel. This trend in telemedicine has not only improved the accessibility of pain management services but also increased patient engagement and satisfaction, aligning with the principles of patient-centered care [61].

Patient-Centered Care in Pain Management

Shared decision-making: Patient-centered care strongly emphasizes shared decision-making, fostering a collaborative partnership between healthcare providers and patients. This approach recognizes that patients have unique perspectives, preferences, and goals for their pain management. Through open and informed discussions, healthcare providers and patients collaborate to select the most appropriate analgesic agents and techniques. This ensures that the chosen pain management plan aligns with the individual patient’s needs and values, enhancing their autonomy and engagement in their care [62].

Pain education: Patient education is a cornerstone of patient-centered care. Healthcare providers take the time to thoroughly inform patients about their pain management options, potential side effects, and the importance of clear and continuous communication. This empowers patients with the knowledge to make informed decisions about their care, fostering a sense of agency and trust in the healthcare provider-patient relationship [63].

Individualized care plans: Patient-centered care recognizes that no two patients are identical. Care plans are individualized to meet patients’ diverse needs, values, and preferences. This means tailoring pain management strategies to each patient’s specific characteristics and circumstances. Individualized care plans ensure that the chosen analgesic agents and techniques are the best fit for the patient, optimizing the effectiveness of pain relief [64].

Quality of life and functional outcomes: Beyond pain relief, patient-centered care takes a holistic approach to care. It considers the broader impact of pain on a patient’s life, including their overall quality of life and functional outcomes. Healthcare providers work to enhance not only the absence of pain but also the presence of well-being, ensuring that patients can return to their daily activities and emotional well-being. This comprehensive perspective acknowledges that pain management is about alleviating pain and improving the patient’s overall experience and recovery [53].

## Conclusions

Adequate postoperative analgesia in orthopedic practice is indispensable for enhancing patient outcomes and ensuring a positive surgical experience. This comprehensive review has elucidated the intricate facets of pain management in orthopedic surgery, emphasizing the significance of optimal pain control in promoting patient comfort, early mobilization, and rapid recovery. Multimodal analgesia, with its diverse range of analgesic agents and techniques, offers a balanced approach to pain management, reducing reliance on opioids and minimizing the associated risks. Moreover, emerging trends and innovations, such as novel analgesic agents, advanced pain assessment methods, and patient-centered care, pave the way for further advancements in the field. Encouraging ongoing research, collaboration, and development is imperative to continually improve pain management in orthopedic practice, ultimately leading to better patient care and enhanced postoperative outcomes.

## References

[1] Sampognaro G, Harrell R (2023). Multimodal Postoperative Pain Control After Orthopaedic Surgery. https://pubmed.ncbi.nlm.nih.gov/34283438/.

[2] Gupta A, Kaur K, Sharma S, Goyal S, Arora S, Murthy RS (2010). Clinical aspects of acute post-operative pain management & its assessment. J Adv Pharm Technol Res.

[3] Abebe MM, Arefayne NR, Temesgen MM, Admass BA (2022). Evidence-based perioperative pain management protocol for day case surgery in a resource limited setting: systematic review. Ann Med Surg (Lond).

[4] Hyland SJ, Wetshtein AM, Grable SJ, Jackson MP (2022). Acute pain management pearls: a focused review for the hospital clinician. Healthcare (Basel).

[5] Garimella V, Cellini C (2013). Postoperative pain control. Clin Colon Rectal Surg.

[6] Alami S, Desjeux D, Lefèvre-Colau MM, Boisgard AS, Boccard E, Rannou F, Poiraudeau S (2011). Management of pain induced by exercise and mobilization during physical therapy programs: views of patients and care providers. BMC Musculoskelet Disord.

[7] Queremel Milani DA, Davis DD (2023). Pain Management Medications. https://pubmed.ncbi.nlm.nih.gov/32809527/.

[8] Buli B, Gashaw A, Gebeyehu G, Abrar M, Gerbessa B (2022). Patient satisfaction with post-operative pain management and associated factors among surgical patients at Tikur Anbessa Specialized Hospital: cross-sectional study. Ann Med Surg (Lond).

[9] Anastase DM, Cionac Florescu S, Munteanu AM, Ursu T, Stoica CI (2014). Analgesic techniques in hip and knee arthroplasty: from the daily practice to evidence-based medicine. Anesthesiol Res Pract.

[10] Chau DL, Walker V, Pai L, Cho LM (2008). Opiates and elderly: use and side effects. Clin Interv Aging.

[11] Banaś K, Więch P, Trojnar P (2022). Selected factors influencing the intensity of postoperative pain in patients after orthopedic and gynecological surgeries. Medicina (Kaunas).

[12] Savage SR, Kirsh KL, Passik SD (2008). Challenges in using opioids to treat pain in persons with substance use disorders. Addict Sci Clin Pract.

[13] Chunduri A, Aggarwal AK (2022). Multimodal pain management in orthopedic surgery. J Clin Med.

[14] Varrassi G, Coluzzi F, Guardamagna VA, Puntillo F, Sotgiu G, Vellucci R (2021). Personalizing cancer pain therapy: insights from the rational use of analgesics (RUA) group. Pain Ther.

[15] Kaye AD, Urman RD, Rappaport Y (2019). Multimodal analgesia as an essential part of enhanced recovery protocols in the ambulatory settings. J Anaesthesiol Clin Pharmacol.

[16] Anekar AA, Hendrix JM, Cascella M (2023). WHO Analgesic Ladder. https://pubmed.ncbi.nlm.nih.gov/32119322/.

[17] Sugai DY, Deptula PL, Parsa AA, Don Parsa F (2013). The importance of communication in the management of postoperative pain. Hawaii J Med Public Health.

[18] Wells N, Pasero C, McCaffery M (2008). Improving the quality of care through pain assessment and management. Patient Safety and Quality: An Evidence-Based Handbook for Nurses.

[19] Gunaydin C, Bilge SS (2018). Effects of nonsteroidal anti-inflammatory drugs at the molecular level. Eurasian J Med.

[20] Ayoub SS (2021). Paracetamol (acetaminophen): a familiar drug with an unexplained mechanism of action. Temperature (Austin).

[21] Pathan H, Williams J (2012). Basic opioid pharmacology: an update. Br J Pain.

[22] Edinoff AN, Kaplan LA, Khan S (2021). Full opioid agonists and tramadol: pharmacological and clinical considerations. Anesth Pain Med.

[23] Cofano S, Yellon R (2023). Hydrocodone. https://pubmed.ncbi.nlm.nih.gov/30725973/.

[24] Reyes Fernandez PC, Wright CS, Warden SJ, Hum J, Farach-Carson MC, Thompson WR (2022). Effects of gabapentin and pregabalin on calcium homeostasis: implications for physical rehabilitation of musculoskeletal tissues. Curr Osteoporos Rep.

[25] Moraczewski J, Awosika AO, Aedma KK (2023). Tricyclic Antidepressants. https://pubmed.ncbi.nlm.nih.gov/32491723/.

[26] Rogawski MA, Löscher W, Rho JM (2016). Mechanisms of action of antiseizure drugs and the ketogenic diet. Cold Spring Harb Perspect Med.

[27] Olawin AM, M Das J (2023). Spinal Anesthesia. https://pubmed.ncbi.nlm.nih.gov/30725984/.

[28] Karcz M, Papadakos PJ (2013). Respiratory complications in the postanesthesia care unit: a review of pathophysiological mechanisms. Can J Respir Ther.

[29] Chang A, Dua A, Singh K, White BA (2023). Peripheral Nerve Blocks. https://pubmed.ncbi.nlm.nih.gov/29083659/.

[30] Pehora C, Pearson AM, Kaushal A, Crawford MW, Johnston B (2017). Dexamethasone as an adjuvant to peripheral nerve block. Cochrane Database Syst Rev.

[31] Sharma V, Morgan PM, Cheng EY (2009). Factors influencing early rehabilitation after THA: a systematic review. Clin Orthop Relat Res.

[32] Shah N, Padalia D (2023). Intrathecal Delivery System. https://pubmed.ncbi.nlm.nih.gov/30855825/.

[33] Bhatia G, Lau ME, Koury KM, Gulur P (2013). Intrathecal drug delivery (ITDD) systems for cancer pain. F1000Res.

[34] Pastino A, Lakra A (2023). Patient-Controlled Analgesia. https://pubmed.ncbi.nlm.nih.gov/31869074/.

[35] Schwenk ES, Mariano ER (2018). Designing the ideal perioperative pain management plan starts with multimodal analgesia. Korean J Anesthesiol.

[36] Bohringer C, Astorga C, Liu H (2020). The benefits of opioid free anesthesia and the precautions necessary when employing it. Transl Perioper Pain Med.

[37] Manworren RC (2015). Multimodal pain management and the future of a personalized medicine approach to pain. AORN J.

[38] Folino TB, Mahboobi SK (2023). Regional Anesthetic Blocks. https://pubmed.ncbi.nlm.nih.gov/33085385/.

[39] Knotkova H, Pappagallo M (2007). Adjuvant analgesics. Med Clin North Am.

[40] Bhatia A, Buvanendran A (2019). Anesthesia and postoperative pain control-multimodal anesthesia protocol. J Spine Surg.

[41] Fenske JN, Berland DW, Chandran S (2021). Pain Management. https://pubmed.ncbi.nlm.nih.gov/34310088/.

[42] Small C, Laycock H (2020). Acute postoperative pain management. Br J Surg.

[43] Kaye AD, Helander EM, Vadivelu N, Lumermann L, Suchy T, Rose M, Urman RD (2017). Consensus statement for clinical pathway development for perioperative pain management and care transitions. Pain Ther.

[44] Mangoni AA, Jackson SH (2004). Age-related changes in pharmacokinetics and pharmacodynamics: basic principles and practical applications. Br J Clin Pharmacol.

[45] Packiasabapathy S, Sadhasivam S (2018). Gender, genetics, and analgesia: understanding the differences in response to pain relief. J Pain Res.

[46] Ghosh R, Alajbegovic A, Gomes AV (2015). NSAIDs and cardiovascular diseases: role of reactive oxygen species. Oxid Med Cell Longev.

[47] Cohen B, Ruth LJ, Preuss CV (2023). Opioid Analgesics. https://pubmed.ncbi.nlm.nih.gov/29083658/.

[48] Chandok N, Watt KD (2010). Pain management in the cirrhotic patient: the clinical challenge. Mayo Clin Proc.

[49] Spampinato SF, Caruso GI, De Pasquale R, Sortino MA, Merlo S (2020). The treatment of impaired wound healing in diabetes: looking among old drugs. Pharmaceuticals (Basel).

[50] Sastic C (2014). Appropriate assessment of patient medication allergies. Hosp Pharm.

[51] Brockow K, Przybilla B, Aberer W (2015). Guideline for the diagnosis of drug hypersensitivity reactions: S2K-Guideline of the German Society for Allergology and Clinical Immunology (DGAKI) and the German Dermatological Society (DDG) in collaboration with the Association of German Allergologists (AeDA), the German Society for Pediatric Allergology and Environmental Medicine (GPA), the German Contact Dermatitis Research Group (DKG), the Swiss Society for Allergy and Immunology (SGAI), the Austrian Society for Allergology and Immunology (ÖGAI), the German Academy of Allergology and Environmental Medicine (DAAU), the German Center for Documentation of Severe Skin Reactions and the German Federal Institute for Drugs and Medical Products (BfArM). Allergo J Int.

[52] Fink R (2000). Pain assessment: the cornerstone to optimal pain management. Proc (Bayl Univ Med Cent).

[53] Haverfield MC, Giannitrapani K, Timko C, Lorenz K (2018). Patient-centered pain management communication from the patient perspective. J Gen Intern Med.

[54] O'Donnell KF (2015). Preoperative pain management education: a quality improvement project. J Perianesth Nurs.

[55] Hyland SJ, Brockhaus KK, Vincent WR, Spence NZ, Lucki MM, Howkins MJ, Cleary RK (2021). Perioperative pain management and opioid stewardship: a practical guide. Healthcare (Basel).

[56] Yaksh TL, Woller SA, Ramachandran R, Sorkin LS (2015). The search for novel analgesics: targets and mechanisms. F1000Prime Rep.

[57] Janczura M, Sip S, Cielecka-Piontek J (2022). The development of innovative dosage forms of the fixed-dose combination of active pharmaceutical ingredients. Pharmaceutics.

[58] Derry S, Wiffen PJ, Kalso EA (2017). Topical analgesics for acute and chronic pain in adults - an overview of Cochrane Reviews. Cochrane Database Syst Rev.

[59] Leroux A, Rzasa-Lynn R, Crainiceanu C, Sharma T (2021). Wearable devices: current status and opportunities in pain assessment and management. Digit Biomark.

[60] Xu X, Huang Y (2020). Objective pain assessment: a key for the management of chronic pain. F1000Res.

[61] Kichloo A, Albosta M, Dettloff K (2020). Telemedicine, the current COVID-19 pandemic and the future: a narrative review and perspectives moving forward in the USA. Fam Med Community Health.

[62] Légaré F, Adekpedjou R, Stacey D (2018). Interventions for increasing the use of shared decision making by healthcare professionals. Cochrane Database Syst Rev.

[63] Bleicher J, Esplin J, Blumling AN (2021). Expectation-setting and patient education about pain control in the perioperative setting: a qualitative study. J Opioid Manag.

[64] Bolton RE, Bokhour BG, Hogan TP, Luger TM, Ruben M, Fix GM (2020). Integrating personalized care planning into primary care: a multiple-case study of early adopting patient-centered medical homes. J Gen Intern Med.

